# Individual determinants of COVID-19 vaccine hesitancy

**DOI:** 10.1371/journal.pone.0258462

**Published:** 2021-11-17

**Authors:** Philip Gerretsen, Julia Kim, Fernando Caravaggio, Lena Quilty, Marcos Sanches, Samantha Wells, Eric E. Brown, Branka Agic, Bruce G. Pollock, Ariel Graff-Guerrero

**Affiliations:** 1 Campbell Family Mental Health Research Institute, Centre for Addiction and Mental Health, Toronto, Ontario, Canada; 2 Department of Psychiatry, University of Toronto, Toronto, Ontario, Canada; 3 Institute of Medical Science, University of Toronto, Toronto, Ontario, Canada; 4 Krembil Centre for Neuroinformatics, Centre for Addiction and Mental Health, Toronto, Ontario, Canada; 5 Institute for Mental Health Policy Research, Centre for Addiction and Mental Health, Toronto, Ontario, Canada; 6 Dalla Lana School of Public Health, University of Toronto, Toronto, Ontario, Canada; 7 Provincial System Support Program (PSSP), Centre for Addiction and Mental Health, Toronto, Ontario, Canada; Bangalore Baptist Hospital, INDIA

## Abstract

**Background:**

Novel coronavirus disease 2019 (COVID-19) vaccine hesitancy is a barrier to achieving herd immunity, and thus, a prominent public health concern. This study aimed to identify the determinants of COVID-19 vaccine hesitancy based on the World Health Organization’s ‘3Cs’ model (i.e., confidence, complacency, and convenience) in the United States (U.S.) and Canada.

**Methods:**

Data from 7678 adults ages 18 or older were collected from the four most populous U.S. States, specifically New York, California, Florida, and Texas, and from English-speaking Canada at three timepoints, in May and July 2020, and March 2021 using a web-based survey (www.covid19-database.com). Sociodemographic information was collected, and comprehensive psychological assessments were administered. Univariate analyses were performed to identify the individual determinants of vaccine hesitancy, which were categorized as: 1) vaccine confidence, 2) vaccine complacency, 3) sociodemographic, and 4) other psychological factors. A series of models were computed using these categorizations.

**Results:**

Mistrust of vaccine benefit (β(SE) = 0.67(0.01), p<0.001, partial η^2^ = 0.26) and lower perceived seriousness of COVID-19 (β(SE) = 0.68(0.02), p<0.001, partial η^2^ = 0.12) were the principal determinants of vaccine hesitancy. Right-wing political affiliation (β(SE) = 0.32(0.02), p<0.001, partial η^2^ = 0.03), higher risk propensity (β(SE) = 0.24(0.02), p<0.001, partial η^2^ = 0.03), and less negative mental health effects of the COVID-19 pandemic (β(SE) = 0.20(0.01), p<0.001, partial η^2^ = 0.03) were the main sociodemographic and psychological determinants. Other sociodemographic determinants included younger age, women, race, and employment status. Lack of vaccine confidence and complacency explained 38% and 21% of the variance in vaccine hesitancy, respectively; whereas, sociodemographic and psychological determinants explained 13% and 11% of the variance in vaccine hesitancy, respectively.

**Discussion:**

Targeted and tailored public health interventions that enhance the public’s confidence in vaccines and emphasize the risk and seriousness of COVID-19 may address COVID-19 vaccine hesitancy. Efforts directed toward specific marginalized and underserved groups may be required to promote vaccine confidence.

## 1. Introduction

It is estimated that approximately 70% of the population must acquire immunity via natural infection or vaccination to achieve adequate herd immunity to the 2019 novel coronavirus disease (COVID-19) [1]. In addition to the development of a safe and effective vaccine, vaccination hesitancy is a key public health concern, which can be influenced by individual, group and contextual factors [2]. Anti-vaccination sentiment represents a significant hurdle to overcome toward achieving the threshold for herd immunity, with as few as 50% of Americans committed to getting a COVID-19 vaccine prior to their availability [3]. Recent surveys conducted in November and December 2020 found that a quarter of individuals in the U.S. and Canada were hesitant to getting a COVID-19 vaccine [4,5]. The governments and public health authorities around the world have been tasked with the challenge of ensuring adequate vaccine acceptance and thus vaccination coverage to ensure herd immunity is achieved.

The World Health Organization (WHO) Strategic Advisory Group of Experts (SAGE) defines ‘vaccine hesitancy’ as a delay in acceptance or refusal of vaccination despite vaccine availability [6]. Vaccine hesitancy is complex, variable, and context, time and vaccine specific [2] and has primarily been studied in relation to infections typically encountered in childhood and influenza. Vaccine hesitant individuals represent a heterogeneous group in the middle of the continuum ranging from acceptors to complete refusers. The SAGE working group’s confidence, complacency, and convenience (i.e., “3 Cs”) model suggests that vaccine hesitancy emerges when individuals (1) *lack confidence* in the safety and effectiveness of the vaccine and the system recommending and providing it; (2) are *complacent*, in that they do not believe the vaccine-preventable disease is serious, vaccination is not necessarily required to prevent infection and transmission, and that possible consequences outweigh the benefits of any vaccine; and (3) perceive that access to the vaccine is *inconvenient*, uncomfortable or unaffordable [2].

Provided public health authorities address the convenience factor of the 3Cs vaccine hesitancy model by optimizing the affordability, accessibility, health literacy, and delivery of vaccines in a culturally appropriate manner, COVID-19 vaccine hesitancy will likely occur as a result of individuals’ low confidence and complacency in relation to any available COVID-19 vaccines. Given vaccine hesitancy is context, time, and vaccine specific [2], we applied the WHO’s 3C classification of vaccination hesitancy model to COVID-19 vaccines. We aimed to identify the individual determinants associated with COVID-19 vaccine hesitancy, which were categorized as: 1) vaccine complacency, 2) vaccine confidence, 3) sociodemographic, and 4) other psychological factors. We administered a comprehensive survey in adults from the four most populous states in the U.S, specifically New York, California, Florida, and Texas, and across English-speaking Canada. Based on prior surveys on vaccine hesitancy conducted in the U.S. and Canada, we hypothesized a lack of vaccine confidence followed by complacency as the main barriers to vaccine uptake, followed by sociodemographic and other psychological factors [7].

## 2. Methods

### 2.1 Data collection

**Fig 1:**
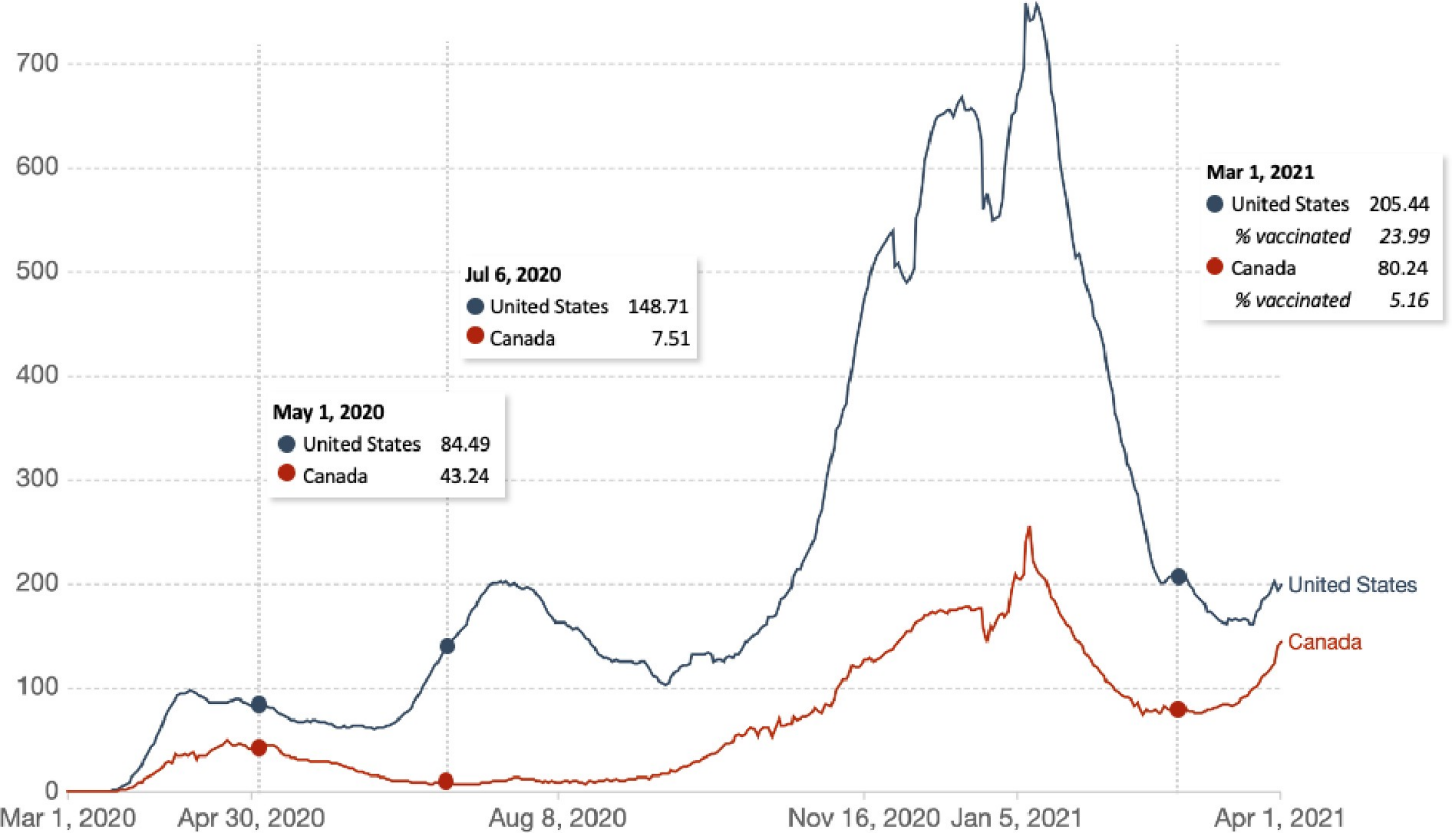
Daily new confirmed COVID-19 cases per million people (rolling 7-day average) in the United States and Canada. The survey data was collected from May 1 to 4, 2020 (n = 1019), July 6 to 10 (n = 3923), and February 12 to March 4, 2021 (n = 2736). Source: COVID-19 Data Repository from the Center for Systems Science and Engineering (CSSE) at Johns Hopkins University via Our World in Data [8].

Data from 7678 participants 18 years of age or older were acquired at three timepoints, in May and July 2020, and March 2021 (**Fig 1**) (http://covid19-database.com/). Different participants were acquired at each timepoint. Our rationale for collecting data at three different timepoints was to assess how vaccine hesitancy changes in relation to the number of COVID-19 cases and vaccine availability. Quotas for age ranges were placed to ensure that data from a representative sample of participants from the U.S. and Canada were collected. We aimed to include approximately an equal number of respondents from the following age ranges: 18–24, 25–34, 35–44, 45–54, 55–64, and 65+ years of age.

Data was collected using a web-based survey platform, *Dynata*, a global market research company (https://www.dynata.com/). Participation in this study was voluntary and open to all participants in the U.S. and in Canada in the specified geographical regions. *Dynata* uses a routing technology that is designed to ensure high-quality sampling. The invitation process involved multiple channels, including email invitations, and banners and messaging on panel community sites. Survey invitations provided basic links to the system, and upon entry, participants were asked additional screening questions to ensure they met the criteria for the study.

Responses were collected from 5038 participants from the four most populous U.S. states, specifically New York (n = 1618, 32%), California (n = 1623, 32%), Florida (n = 899, 18%), and Texas (n = 898, 18%). Responses were also collected from 2640 participants from English-speaking Canada, specifically from Ontario (n = 1267, 48%), British Columbia (n = 515, 20%), and Prairie (n = 611, 23%) and Atlantic provinces (n = 247, 9%).

Survey attempts not included in the study were removed for the following reasons: over quota (n = 111), partial completes (n = 2544), terminated the survey (n = 1093), failed open-end manual checks (n = 631), and completed the survey too quickly (n = 223).

All participants provided written informed consent prior to completing the survey. The study was approved by the Research Ethics Board (REB) of our institution. We followed the EQUATOR Reporting Guidelines.

### 2.2 Measures

Participants’ degree of vaccine hesitancy was assessed using the following question: “Would you get vaccinated if a vaccine for COVID-19 becomes available?” The answer options consisted of a Likert scale, ranging from ‘1, Definitely’ to ‘6, Definitely Not’, with a higher score representing greater vaccine hesitancy.

Participants provided detailed sociodemographic information and completed a battery of assessments, including single-item and multi-item questionnaires to assess the degree of complacency and confidence in relation to COVID-19 and COVID-19 specific vaccines. The following were used to assess complacency: (1) perceived susceptibility to infectious disease using the Perceived Vulnerability to Disease Questionnaire (PVD), the infectability subscale [9], (2) perceived seriousness of COVID-19 and restrictiveness of the current physical (social) distancing restrictions, (3) prior testing for COVID-19 (self and close others) using single-items, and (4) health risk factors for COVID-19. To measure confidence, the following questionnaires were used: (1) vaccine mistrust using the Vaccine Attitude Examination (VAX) scale [10], (2) preference for alternative medicine using the Holistic Complementary and Alternative Medicine Questionnaire (HCAM) [11], and (3) trust in Government’s management of COVID-19 using the Citizen Trust in Government Organization Scale (CTGO) [12].

Additionally, participants completed a battery of other psychological assessments that may contribute to vaccine hesitancy. These included the Ten-Item Personality Inventory (TIPI) [13], general Risk Propensity Scale (RPS) [14], Multidimensional Iowa Suggestibility Scale (MISS) [15], Authority Behavior Index (ABI) [16], General Trust Scale (GTS) [17], Brief Locus-of-Control Scale (LOC) [18], Positive and Negative Affect Schedule (PANAS) [19], and Experiences in Close Relationships (ECR) scale [20], and a single-item to assess the impact of COVID-19 on the participant’s mental health. Information regarding development, recruitment, and quality control measures can be found in **S1 File**.

### 2.3 Statistical analysis

Statistical analyses were performed using IBM SPSS Statistics (v26 IBM Corp., Armonk, N.Y., US). Univariate analyses were performed to examine the associations between vaccine hesitancy and the following four categories of variables: 1) vaccine complacency, 2) vaccine confidence, 3) sociodemographic, and 4) other psychological factors. Separate models were computed for each category and an R^2^ value was derived for each model. Beta (*β* and partial eta squared (η^2^) values for each variable were derived and a threshold of p<0.006 was used to determine significance (0.05/8 models). Partial η^2^ values were used to define small (η^2^ = 0.01), medium (η^2^ = 0.06), and large (η^2^ = 0.14) effects [21,22]. The analyses were repeated, separately controlling for sociodemographic factors, timepoint (i.e., survey completed in May 2020, July 2020 or March 2021), and COVID-19 vaccination status (i.e., vaccinated or not vaccinated). A final analysis was performed including all of the categories in a single model to determine the total variance explained by the categories.

## 3. Results

### 3.1 Participant characteristics

The sociodemographic and clinical characteristics of the 7678 participants included in the study are listed in **Table 1**. Participants were broadly representative of the U.S. and Canadian population with respect to age (mean = 47.2±17.3) and gender (50.8% woman). The majority of participants identified their race as White (68.3%). One percent of the participants were Indigenous (Native American and Indigenous People of Canada including First Nations, Inuit, and Métis), 4.6% Black, 9.5% East Asian, 7.1% Latinx, 2.5% South Asian, and 7.0% indicated ‘other’. A large proportion of our sample identified with a religion (67.5%), with the greatest representation being Christians (45%), the majority of which endorsed being Roman Catholic (26.1%). Thirty-one percent of our sample reported having ‘no religion’.

**Table 1:** Participant characteristics (N = 7678).

	Mean (SD), range *or* N (%)
Age	47.2 (17.3)
Gender (man/woman)	3762 (49.2)/3883 (50.8)
Education	
Some high school or less	132 (1.7)
Completed high school	911 (11.9)
Some college/university	1239 (16.1)
Completed college/university	3677 (47.9)
Post graduate or higher	1719 (22.4)
Race	
Indigenous (First Nations, Inuit or Métis)	78 (1.0)
Black	355 (4.6)
East Asian	728 (9.5)
Latinx	544 (7.1)
White	5243 (68.3)
South Asian	191 (2.5)
Other	539 (7.0)
Religion (yes/no)	4948 (67.5)/2384 (32.5)
Canada/United States	2640 (34.4)/5038 (65.6)
State	
New York	1618 (32.1)
California	1623 (32.2)
Florida	899 (17.8)
Texas	898 (17.8)
Province	
Atlantic provinces	247 (9.4)
Ontario	1267 (48.0)
Prairie provinces	611 (23.1)
British Columbia	515 (19.5)
Population size	
1,000 or less	204 (3.0)
1,000 to 29,999	777 (11.3)
30,000 to 99,999	1244 (18.1)
100,000 or more	4635 (67.6)
Household income	
less than $20,000	499 (6.9)
$20,000 - $59,999	1877 (26.1)
$60,000 - $99,999	2073 (28.8)
$100,000 - $139,999	1292 (18.0)
$140,000 or more	1450 (20.2)
Employment status	
Unemployed	888 (11.6)
Employed	4377 (57.0)
Student	400 (5.2)
Retired	1635 (21.3)
Other	378 (4.9)
Healthcare worker (yes/no)	1107 (14.2)/6675 (85.8)
Political spectrum	
Communism left wing or socialism	435 (5.7)
Liberal	2193 (28.6)
Center	2808 (36.6)
Conservative	2081 (27.1)
Fascism right wing or authoritarianism	161 (2.1)
COVID-19 health risk factors	
COVID-19 health risk factor score^a^	0.7 (1.1), 0–8
Heart disease (yes/no)	498 (5.2)/7380 (94.8)
Hypertension (yes/no)	1699 (22.1)/5979 (77.9)
Lung disease (yes/no)	285 (3.7)/7393 (96.3)
Diabetes (yes/no)	935 (12.0)/6753 (88.0)
Cancer (yes/no)	250 (3.3)/7428 (96.7)
Chronic kidney disease (yes/no)	126 (1.6)/7552 (98.4)
Obesity (yes/no)	977 (12.7)/6701 (87.3)
Weakened immune system (yes/no)	721 (9.4)/6957 (90.6)

^a^One point was assigned for each health risk factor (i.e., heart disease, hypertension, lung disease, diabetes, cancer, chronic kidney disease, obesity, and weakened immune system) to derive a total health risk factor score for COVID-19.

With respect to political affiliation, 5.7% of the participants indicated communist left wing or socialist, 28.6% liberal, 36.6% center, 27.1% conservative, and 2.1% fascist right wing or authoritarian.

The most frequently reported household income was $60,000 to $99,999 (28.8%) and the majority of the participants were employed (57.0%), however, 11.6% of the participants were unemployed. Students and retirees represented 5.2% and 21.3% of the sample, respectively.

### 3.2 Vaccination hesitancy

**Fig 2:**
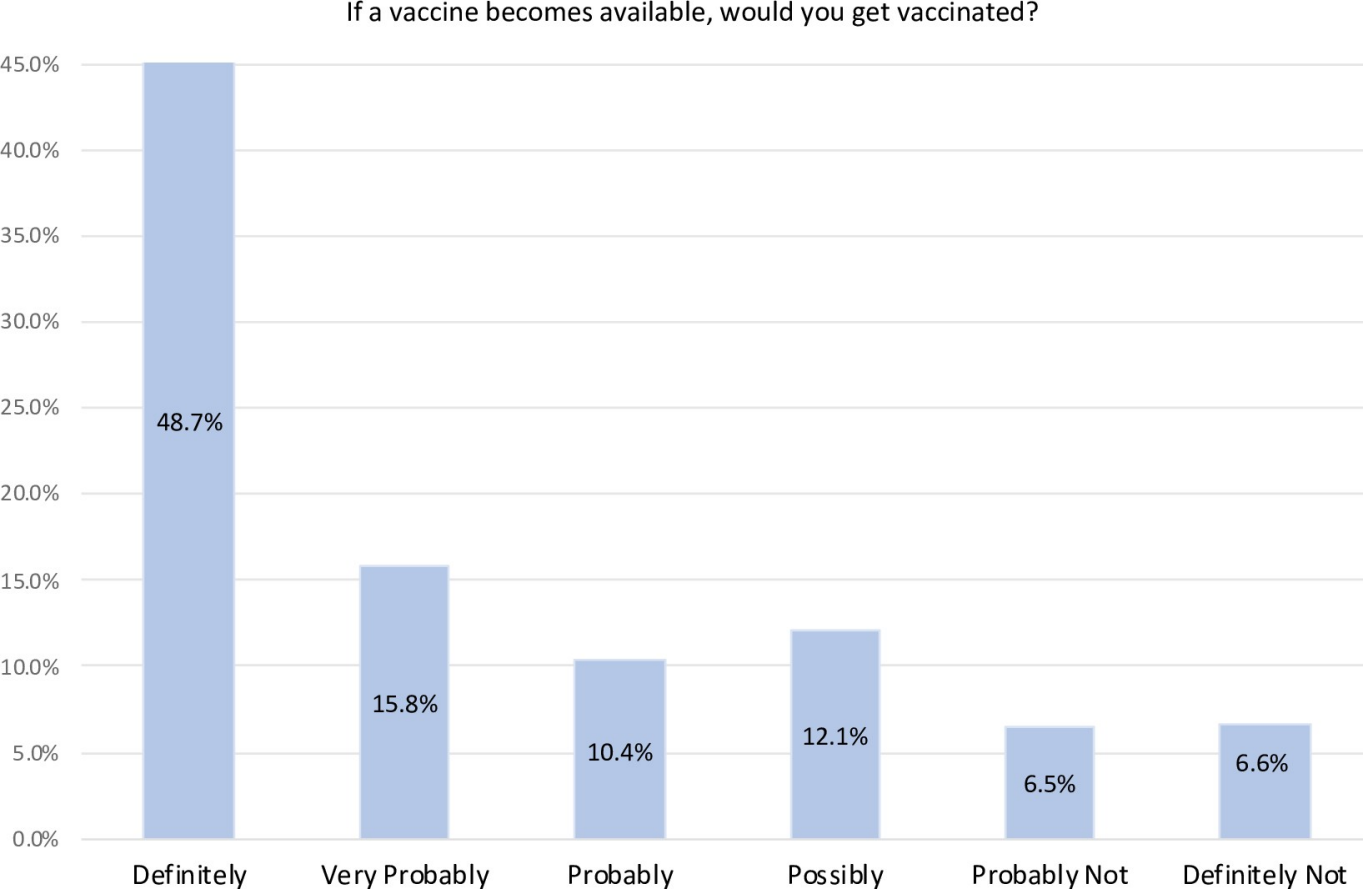
COVID-19 vaccine hesitancy distribution in the United States and Canada (n = 7678).

**Fig 2** shows the distribution in the degree of COVID-19 vaccine hesitancy. The mean (SD) hesitancy scores were 2.3/6.0 (1.6), which corresponds to 74.9% of the sample ‘probably’ to ‘definitely’ likely to get vaccinated if a COVID-19 was available. At the time of the survey in March 2021, 2.7% of the participants in Canada (n = 19/704) were vaccinated for COVID-19 compared to 29.5% in the United States (n = 600/2032). Vaccine hesitancy was not significantly different between the three timepoints (F(2,7677) = 3.45, p = 0.032).

### 3.3 Sociodemographic factors associated with vaccine hesitancy

Sociodemographic factors associated with vaccine hesitancy included younger age, women, race (i.e., Black participants), employment status (i.e., employed compared to retirees), and right-wing political status (R^2^ = 0.13, F(36,7504) = 31.47, p<0.001) (**Table 2**). Controlling for timepoint did not significantly change the results. Lower population density (*β*(SE) = 0.34 (0.08), p<0.001, partial η^2^ = 0.01) and lower household income (*β*(SE) = 0.53 (0.13), p<0.001, partial η^2^ = 0.01) emerged as main determinants of vaccine hesitancy when controlling for vaccination status.

**Table 2:** Univariate analyses examining the relationship between COVID-19 vaccine hesitancy and sociodemographic, complacency, confidence, and other psychological factors.

	Beta	SE	t	p-value	Partial η2
Sociodemographic factors	(R^2^: 0.13)				
Age	-0.01	0.00	-6.85	<0.001*	0.01^1^
Gender (man/woman^a^)	-0.22	0.04	-6.15	<0.001*	0.01^1^
Education (years)					
Some high school or less	0.53	0.14	3.67	<0.001*	0.00
Completed high school	0.27	0.07	3.99	<0.001*	0.00
Some college/university	0.21	0.06	3.36	0.001*	0.00
Completed college/university	0.12	0.05	2.61	0.009*	0.00
Post graduate or higher^a^	-	-	-	-	-
Race					
Indigenous (First Nations, Inuit or Métis)	0.54	0.17	3.15	0.002*	0.00
Black	0.94	0.09	10.97	<0.001*	0.02^1^
East Asian	0.05	0.06	0.84	0.402	0.00
Latinx	0.11	0.07	1.57	0.116	0.00
South Asian	0.02	0.11	0.13	0.896	0.00
Other	0.28	0.07	3.98	<0.001*	0.00
White^a^	-	-	-	-	-
Religion (yes/no^a^)	-0.02	0.04	-0.51	0.609	0.00
Region (states and provinces)					
California	-0.07	0.06	-1.16	0.248	0.00
Florida	0.10	0.07	1.42	0.155	0.00
New York	-0.07	0.06	-1.19	0.234	0.00
Texas	-0.01	0.07	-0.19	0.853	0.00
Atlantic Provinces	-0.24	0.11	-2.20	0.028	0.00
Prairie Provinces	-0.04	0.08	-0.50	0.615	0.00
British Columbia	-0.05	0.08	-0.57	0.570	0.00
Ontario^a^	-	-	-	-	-
Population density					
1,000 or less	0.39	0.11	3.61	<0.001*	0.00
1,000 to 29,999	0.14	0.06	2.40	0.016	0.00
30,000 to 99,999	0.21	0.05	4.31	<0.001*	0.00
100,000 or more^a^	-	-	-	-	-
Political affiliation	0.32	0.02	16.32	<0.001*	0.03^1^
Healthcare worker status (yes/no^a^)	0.02	0.05	0.32	0.749	0.00
Employment status					
Unemployed	-0.06	0.06	-0.98	0.327	0.00
Employed^a^	-	-	-	-	-
Student	-0.24	0.09	-2.80	0.005*	0.00
Retired	-0.40	0.06	-6.70	<0.001*	0.01^1^
Household income					
Less than $20,000	0.37	0.08	4.59	<0.001*	0.00
$20,000-$59,999	0.28	0.05	5.61	<0.001*	0.00
$60,000-$99,999^a^	-	-	-	-	-
$100,000-$139,999	-0.07	0.05	-1.35	0.178	0.00
$140,000 or more	-0.24	0.05	-4.46	<0.001*	0.00
**Complacency factors**	(R^2^: 0.21)				
Perceived susceptibility to infectious disease	-0.05	0.02	-2.94	0.003*	0.00
Perceived seriousness of COVID-19	-0.68	0.02	-28.01	<0.001*	0.12^2^
Perceived safety of social distancing measures	0.00	0.02	0.17	0.863	0.00
Perceived safety of going out in the community	0.04	0.02	2.34	0.020	0.00
Perceived likelihood of more waves of COVID-19	-0.11	0.02	-4.63	<0.001*	0.00
Tested positive for COVID-19 (self) (Tested positive/ Not tested or tested negative^a^)	-0.07	0.04	-1.81	0.071	0.00
Tested positive for COVID-19 (someone close) (Tested positive/ Not tested or tested negative^a^)	-0.21	0.04	-4.80	<0.001*	0.00
COVID-19 health risk factors^b^	-0.11	0.02	-6.66	<0.001*	0.01^1^
**Confidence factors**	(R^2^: 0.38)				
Mistrust of vaccine benefit	0.67	0.01	51.64	<0.001*	0.26^3^
Worries over unforeseen future effects	0.04	0.02	2.58	0.010	0.00
Concerns about commercial profiteering	0.11	0.02	7.06	<0.001*	0.01^1^
Preference for natural immunity	0.11	0.02	7.38	<0.001*	0.01^1^
Positive attitudes toward holistic health approaches	-0.01	0.00	-2.59	0.010	0.00
Positive attitudes toward complementary and alternative medicine	-0.01	0.00	-3.00	0.003*	0.00
Mistrust in Government’s management of COVID-19	0.01	0.00	5.74	<0.001*	0.00
**Other psychological factors**	(R^2^: 0.11)				
TIPI, Extraversion	0.03	0.02	1.76	0.079	0.00
TIPI, Agreeable	0.06	0.02	2.92	0.004*	0.00
TIPI, Conscientiousness	-0.03	0.02	-1.52	0.128	0.00
TIPI, Emotional stability	-0.05	0.02	-2.37	0.018	0.00
TIPI, Openness to experience	-0.03	0.02	-1.55	0.121	0.00
RPS, Risk propensity	0.24	0.02	14.16	<0.001*	0.03^1^
MISS, Suggestibility	0.00	0.00	-1.21	0.226	0.00
Attitudes toward authority	0.00	0.00	-1.57	0.116	0.00
General trust in others	-0.30	0.03	-10.88	<0.001*	0.02^1^
LOC, Internal	-0.01	0.01	-1.99	0.047	0.00
LOC, Chance	-0.03	0.01	-4.06	<0.001*	0.00
LOC, Powerful others	0.01	0.01	1.96	0.050	0.00
PANAS, Positive affect score	-0.01	0.00	-2.67	0.008*	0.00
PANAS, Negative affect score	0.02	0.00	4.76	<0.001*	0.00
ECR, Attachment anxiety subscale	0.00	0.00	0.88	0.377	0.00
ECR, Attachment avoidance subscale	0.00	0.00	0.78	0.437	0.00
Impact of COVID-19 on mental health	-0.20	0.01	-15.39	<0.001*	0.03^1^

SE, Standard Error; TIPI, Ten-Item Personality Inventory; RPS, Risk Propensity Scale; MISS, Multidimensional Iowa Suggestibility Scale; LOC, Brief Locus-of-Control Scale; PANAS, Positive and Negative Affect Schedule; ECR, Experiences in Close Relationship.

^a^Reference variable.

^b^One point was assigned for each health risk factor (i.e., heart disease, hypertension, lung disease, diabetes, cancer, chronic kidney disease, obesity, and weakened immune system) to derive a total health risk factor score for COVID-19.

*p<0.010 (0.05/4 models); ^1^Small effect (η^2^ = 0.01); ^2^Medium effect (η^2^ = 0.06); ^3^Large effect (η^2^ = 0.14).

### 3.4 Complacency factors associated with vaccine hesitancy

Complacency factors associated with vaccine hesitancy included lower perceived seriousness of COVID-19 and less health risk factors for COVID-19 (R^2^ = 0.21, F(8,5733) = 189.90, p<0.001) (**Table 2**). Controlling for timepoint did not significantly change the results. Lower perceived susceptibility to infectious disease emerged as one of the main determinants of vaccine hesitancy when controlling for sociodemographic factors (*β*(SE) = 0.10 (0.02), p<0.001, partial η^2^ = 0.01). Health risk factors for COVID-19 was no longer a determinant when controlling for sociodemographic factors or vaccination status.

### 3.5 Confidence factors associated with vaccine hesitancy

Confidence factors associated with vaccine hesitancy included mistrust in vaccine benefit, concerns about commercial profiteering, and preference for natural immunity (R^2^ = 0.38, F(7,7670) = 684.10, p<0.001) (**Table 2**). When controlling for sociodemographic factors, mistrust in the government’s management of COVID-19 emerged as one of the determinants (*β*(SE) = 0.02 (0.00), p<0.001, partial η^2^ = 0.01), while concerns about commercial profiteering was no longer a determinant. The results did not change when controlling for timepoint or vaccination status.

### 3.6 Psychological factors associated with vaccine hesitancy

Other psychological factors associated with vaccine hesitancy included risk propensity, general mistrust in others, and negative impact of COVID-19 on mental health (R^2^ = 0.11, F(17,6641) = 47.92, p<0.001) (**Table 2**). The results remained the same when controlling for sociodemographic factors, timepoint or vaccination status.

### 3.7 Single model of vaccine hesitancy: Sociodemographic, psychological, complacency, and confidence factors

**Fig 3:**
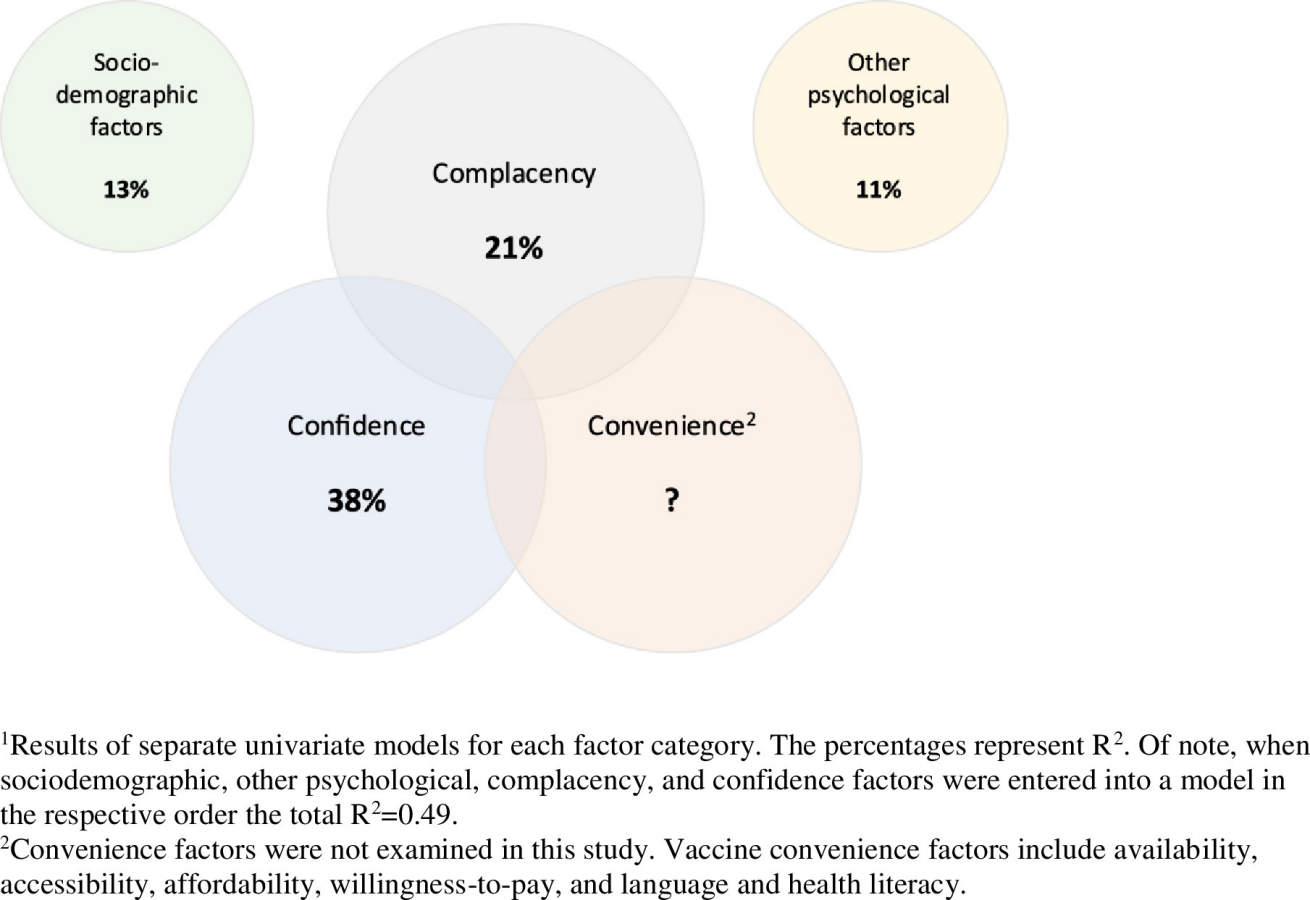
Variance of COVID-19 vaccine hesitancy explained by vaccine confidence, vaccine complacency, sociodemographic, and other psychological factors^1^.

When entered into a single model in the following order: sociodemographic, psychological, complacency, and confidence factors, the total variance explained by the factors was 49.2% (F(68,5651) = 82.43, p<0.001). **Fig 3** shows the variance of COVID-19 vaccine hesitancy explained by sociodemographic, complacency, confidence, and other psychological factors.

## 4. Discussion

Addressing vaccine hesitancy is essential to achieving herd immunity via vaccination in a timely manner to minimize the morbidity and mortality associated with the natural spread of COVID-19. From a public health perspective, as of July 2020 (the date of the last data collection), 74.9% of U.S. and Canadian adults were “probably” to “definitely” likely to get a COVID-19 vaccine, if deemed effective and available. These results are consistent with a recent study exploring the impact of public perception of vaccine efficacy and safety on the likelihood of U.S. and Canadian adults accepting a COVID-19 vaccination [23].

This study identified the individual determinants of vaccine hesitancy employing the WHO SAGE working group’s ‘3Cs’ model of vaccine hesitancy (i.e., Confidence, Complacency, and Convenience), with a focus on confidence and complacency. The results of our study identified vaccine mistrust, followed by perceived seriousness of COVID-19 as the main individual determinants of vaccine hesitancy. To a lesser degree, sociodemographic and other psychological factors were also significant contributors to vaccine hesitancy (**Fig 3**). The degree to which vaccine convenience will contribute to the acceptance of COVID-19 vaccines remains to be determined as this was beyond the scope of the present study. To optimize vaccine convenience, governments and public health authorities are tasked with ensuring vaccines, when available, are easily accessible, affordable, and delivered in a comfortable and culturally sensitive manner [2].

Vaccine confidence explained 38% of the variance of vaccine hesitancy. The main determinant of vaccine confidence was mistrust in vaccine benefit. Other significant factors, to a much lesser degree, were concerns about commercial profiteering, preference for natural immunity, and mistrust in government’s management of COVID-19. Lack of vaccine confidence, in particular mistrust in the safety and efficacy of a COVID-19 vaccine, represents a significant barrier to vaccine acceptance toward achieving the threshold for herd immunity [3], but one which could be addressed by health education campaigns. Multi-component public health strategies are likely required to address the inevitable emergence of vaccine misinformation via social media specific to any novel COVID-19 vaccine [24].

Vaccine complacency explained 21% of the variance of COVID-19 vaccine hesitancy. The principal determinant of vaccine complacency was lower perceived seriousness of COVID-19. These results suggest that strategic public health messaging targeting individuals currently minimizing the seriousness of COVID-19 will be required to address vaccine hesitancy in this group. Future studies may benefit from qualitative methods to identify the factors that contribute to the perceived seriousness of COVID-19, which likely include perceptions of the risk of transmission and the likelihood of serious illness and associated morbidity.

Sociodemographic factors explained 13% of the variance of COVID-19 vaccine hesitancy. The main sociodemographic determinant of vaccine hesitancy was right-wing political ideology. By comparison, in a COVID-19 survey conducted in France, participants that voted either for a far left or far right candidate were more likely to refuse a future COVID-19 vaccine [25]. Other significant sociodemographic factors were younger age, women, race (i.e., Black compared to White participants), and employment status (i.e., employed compared to retirees). Lower population density and lower household income emerged as determinants of vaccine hesitancy when controlling for vaccination status. Together, these sociodemographic determinants indicate that marginalized groups in terms of gender, race, rurality and income are most hesitant to receive a COVID-19 vaccine and are consistent with another recent study that found an association between many of these determinants and vaccine hesitancy [23]. Psychological factors explained 11% of the variance of COVID-19 vaccine hesitancy. Risk propensity, less general trust in others, and less negative mental health effects of COVID-19 were the main psychological determinants of vaccine hesitancy.

There are a few limitations to this study. First, using a web-based survey will not capture responses from individuals who do not have access to or familiarity with using a computer. Second, our participant sample may be biased in that individuals that participate in research studies are more likely to have certain personality characteristics, including conscientiousness and agreeableness [26]. The advantage of using web-based surveys is that they provide a platform to reach a large number of participants within a short period with high validity and reliability that were traditionally collected in-person or via the traditional postal system with high validity and reliability [27]. Third, we are unable to comment on the direction of the associations as our data is cross-sectional. Fourth, only completed survey data were considered. Therefore, we are unable to compare the participant characteristics and responses between included and non-included survey attempts. Last, data collected in May and July 2020 was prior to vaccine availability for COVID-19 [28].

## 5. Conclusions

Encouragingly, at the time of this study, there remained 22.5% of individuals who would ‘possibly’ or ‘probably’ get vaccinated once available, highlighting the need for public health authorities to develop interventions to pre-emptively reduce vaccine hesitancy to achieve herd immunity. Instilling trust in vaccine benefit and emphasizing the risks of COVID-19 will target the main determinants of vaccine confidence and complacency, respectively. Public health interventions tailored to specific marginalized and underserved communities are required to promote vaccine confidence, including women, specific racial groups, and those with lower income and from smaller population centers. To ensure vaccine convenience is optimized, COVID-19 vaccines must be easily accessible, affordable, and delivered in a comfortable and culturally sensitive manner [2].

## Supporting information

S1 File Development and pre-testing of the survey, recruitment process, and quality control measure. (DOCX)
