# Supplementary material for: Individual determinants of COVID-19 vaccine hesitancy
Source: PLoS One. 2021 Nov 17;16(11):e0258462. doi: 10.1371/journal.pone.0258462 (PMC8598046; doi:10.1371/journal.pone.0258462)
Supplement: S1 File — (DOCX) [file pone.0258462.s001.docx]

**Supplemental Material 1.** Development and pre-testing of the survey, recruitment process, and quality control measure.

**Development and pre-testing**

The survey was tested by our team (PG, JK, and AG) to ensure usability and technical functionality. The soft launch data were inspected by Dynata and our team prior to the hard launch.

**Recruitment process and survey administration**

Participants were invited by e-mail based on their demographic profile to obtain a sample representative of the adult population in the United States and Canada. The survey was quota controlled for age and region. The email invitation read “Start the survey and voice your opinions.” Participants were also able to take the survey by accessing Dynata’s survey platform. Participants received panel credit points for their time and participation.

The order of some questions was randomized to prevent response bias. A number of questions were asked conditionally to reduce the number and complexity of the questions (e.g., ‘branching logic’ was used where possible). A maximum of one questionnaire was asked per screen. The number of questions per page ranged from 1 to a maximum of 24. Participants’ answers were considered final if they proceed to the next screen.

**Quality control**

Quality control measures were placed, including manual open end verbatim and racer checks. Data from participants who completed the survey in less than 30% of the median survey length were removed. IP address and digital fingerprints were used to prevent respondents from completing the survey more than once.

**Survey response**

Survey attempts not included in the study were removed for the following reasons: over quota (n=111), partial completes (n=2544), terminated the survey (n=1093), failed open-end manual checks (n=631), and completed the survey too quickly (n=223).
